# Repository of Enriched Structures of Proteins Involved in the Red Blood Cell Environment (RESPIRE)

**DOI:** 10.1371/journal.pone.0211043

**Published:** 2019-02-22

**Authors:** S. Téletchéa, H. Santuz, S. Léonard, C. Etchebest

**Affiliations:** 1 Institut National de la Transfusion Sanguine, Paris, France; 2 Inserm, UMR_S 1134, Paris, France; 3 Université Paris Diderot, Sorbonne Paris Cité, Paris, France; 4 Laboratory of Excellence GR-Ex., Paris, France; 5 UFIP, University of Nantes, CNRS UMR 6286, Nantes, France; Linköping University, SWEDEN

## Abstract

The Red Blood Cell (RBC) is a metabolically-driven cell vital for processes such a gas transport and homeostasis. RBC possesses at its surface exposing antigens proteins that are critical in blood transfusion. Due to their importance, numerous studies address the cell function as a whole but more and more details of RBC structure and protein content are now studied using massive state-of-the art characterisation techniques. Yet, the resulting information is frequently scattered in many scientific articles, in many databases and specialized web servers. To provide a more compendious view of erythrocytes and of their protein content, we developed a dedicated database called RESPIRE that aims at gathering a comprehensive and coherent ensemble of information and data about proteins in RBC. This cell-driven database lists proteins found in erythrocytes. For a given protein entry, initial data are processed from external portals and enriched by using state-of-the-art bioinformatics methods. As structural information is extremely useful to understand protein function and predict the impact of mutations, a strong effort has been put on the prediction of protein structures with a special treatment for membrane proteins. Browsing the database is available through text search for reference gene names or protein identifiers, through pre-defined queries or via hyperlinks. The RESPIRE database provides valuable information and unique annotations that should be useful to a wide audience of biologists, clinicians and structural biologists.

**Database URL:** http://www.dsimb.inserm.fr/respire

## Introduction

Blood is essential to life for (i) the transportation of oxygen and carbon dioxide alongside metabolites, cells and nutrients, (ii) for homeostasis by participating in temperature regulation and pH maintenance, and (iii) for blood vessel protection from injury via platelet aggregation. In volume, blood is composed of about 55% plasma containing water, proteins, electrolytes, glucose and amino acids and about 45% of red blood cells (RBCs), known as erythrocytes. The red blood cells derive from hematopoietic stem cells that undergo several differentiation steps [1,2] leading to cells void of organelles, of protein synthesis material and of nuclear DNA. The main protein found in RBC is haemoglobin, involved in oxygen and carbon dioxide fixation and transport. Beside this abundant protein with a vital functional role, strong efforts have been accomplished to identify other RBC proteins alongside the erythropoiesis process [3–14]. Indeed, many other cellular processes also occur in the RBC including ion and metabolites exchange with plasma, which is ensured by specialized membrane protein complexes. These membrane complexes may also carry specific epitopes (characterizing the so-called “blood groups”), which are vital for blood transfusion.

Many attempts have been made to assemble and organize the existing knowledge on genes and proteins important in RBC in open/free dedicated databases (**Table 1**). In these databases, gene or protein-driven, sometimes genetic, clinical or genomic information is provided. It is however difficult to gather a cell-centric view of the RBC protein content in different conditions, for instance how variants of a given protein will be linked to new RBC group antigens definitions [15,16] or alternatively how mutations can be related to diseases from diverse origins [17]. Consequently, in order to obtain a more comprehensive view of the human RBC content and its implications in physiological and pathological conditions [18,19], we have set up a dedicated database that aims at gathering in a one-stop window crucial information related to RBC. This database is called the **Repository of enriched structures of proteins involved in the red blood cell environment (RESPIRE)**.

**Table 1:** Databases providing access to red blood cells gene and protein expression.

Database name and URL	Description	Reference
BloodSpot, http://servers.binf.ku.dk/bloodspot/	Gene expression in mice and human hematopoiesis in normal and pathological conditions	[20]
Erythrogene, http://www.erythrogene.com/	Genetic variation of the 36 blood group antigens extracted from the 1000 genomes project	[21]
HbVar, http://globin.cse.psu.edu/hbvar	Database linking haemoglobin genomic mutations with thalassemia and hemoglobinopathies	[22]
Red Blood Cell Collection, http://rbcc.hegelab.org/	Compendium of proteins detected in red blood cells for which their presence is qualified by a confidence index	[5]
The Human RhesusBase, http://rhesusbase.info/	This database integrates a very up-to-date knowledge on the rhesus locus and its consequence to the RH antigen D expression and phenotype	[23] (Note that Respire Database is cited as related resources in this database)

The red blood cell protein content was extracted from review of the erythrocyte content performed by Goodman and co-workers [24] and completed with very recent proteomics analyses [25,26] when list of protein identifiers were available. We have also added and curated this list with the proteins involved in regular red blood cell antigens definition. Proteins in Goodman’s list that were not identified in subsequent studies were not included in the present version, even though they were submitted to the whole treatment process. The corresponding data are available upon request. Data from external databases were processed for direct display in RESPIRE to limit the burden of gathering different information from various sources. More importantly, besides collecting scattered data from diverse sources, the originality of RESPIRE lies in the additional information it brings. This enrichment is obtained by applying cutting-edge bioinformatics methods resulting in **enriched sequence annotation** but also original **structural data availability**. At different steps, resulting data, *e*.*g*. multiple sequence alignments, can be downloaded for further processing and use. As it is now well admitted that 3D structure is an unavoidable link between sequence and function [27], we chose to provide to the user with as much structural information as possible using current data available or through state-of-the-art structural bioinformatics prediction methods. Interestingly, since RBC membrane proteins play an important role in transfusion and in blood physiology, we paid a specific attention to membrane proteins, particularly to their 3D modelling. In these regards, a main difficulty arose from the distinction between “membrane-associated” protein and fully embedded membrane proteins. The corresponding annotation was mainly retrieved from uniprot or gene ontology databases. Tools to predict membrane protein topology were also used [28]. Results can be provided upon request. These predictions were also useful to build the 3D models. Each protein model can be manipulated and visualized in 3D within RESPIRE without any expert knowledge.

Altogether, this makes RESPIRE a unique resource in RBC knowledge.

The database content is updated monthly, it is possible to follow only one protein entry by subscribing to its RSS feed, otherwise a more general report is indicated in the database history tab.

## Material and methods

### Data sources and preprocessing

Many RBC proteomics studies were performed to list the proteins available in the final stages of the red blood cell differentiation [3,6–11]. These analyses were initially synthesized by Goodman and co-workers in a comprehensive list of proteins published in 2013 [24]. From the gene list presented in their work, a gene to protein mapping was performed using the ID mapping tool available at UNIPROT [29]. This protein list was completed using the red blood cell antigen system referenced in HGNC [30] and ISBT [31]. In addition, we considered a list of proteins recently identified by mass spectrometry analyses [25,26]. However, up-to-date proteomics analyses do not necessarily agree on RBC proteome content. For instance, the number of entries given in [26] and [25] slightly differs (1942 and 1815 respectively), 83% proteins being in common. The overlap with Goodman’s list is even smaller (~70%), which might indicate dubious attribution, even though the compilation was carefully conducted by experts. Thus, in order to be the most exhaustive as possible, we chose to join the three lists. For each entry, we searched for additional works that sustain the attribution and used data provided by 7 publications [5,24–26,32–34]. We then proceeded to a careful curation that consisted in eliminating from Goodman’s list proteins not available in reviewed uniprot entries indicated in the two recent proteomic studies [25,26]. This procedure allowed us to avoid or at least to limit the number of proteins with dubious RBC attribution that may originate from contaminants. For each entry, the “Evidence” tab recapitulates the publications that support their finding. The definitive list in the database consists of 2475 unique proteins. Out of these proteins, according to their UNIPROT annotation, 384 are membrane proteins with a single-pass or a multiple-pass membrane domain (545 in GO annotation), 1190 are cytoplasmic proteins, and 647 proteins are found in the nucleus. Some proteins are found in multiple or smaller compartments, a more complex sub-cellular location decomposition from gene ontology annotation is available on the statistics tab in RESPIRE. A representative indication of data processing for a given protein entry is now described. The incorporation of a protein of interest into the database was initially performed using its UNIPROT identifier from the curated list of proteins. The UNIPROT [29] xml file was retrieved and then processed using biopython [35] to extract reference data altogether with identifiers for NCBI's Reference Sequence [36], PFAM [37], and the Human Gene Nomenclature Committee [30] if they exist. Second, additional information is incorporated: (i) Gene Ontology records [38], (ii) mutations and links to phenotypes when available, (iii) OMIM entries [Online Mendelian Inheritance in Man, OMIM. McKusick-Nathans Institute of Genetic Medicine, Johns Hopkins University (Baltimore, MD), 2014. World Wide Web URL: http://omim.org/], (iv) experimentally determined three-dimensional structures, and (v) binding partners [39]. For membrane proteins, the expert upstream UNIPROT annotations are transferred without modification for transmembrane segments. Data for protein content available along different stages of erythropoiesis characterized using mass spectroscopy experiments were provided by our partner in the GR-Ex consortium [12] (http://www.labex-grex.com)

### Sequence conservation, coevolution and analysis

Besides the human sequence, which constitutes the main purpose of RESPIRE, we chose to gain information about the protein family. For this purpose, (i) a BLAST [40] search was performed to retrieve sequences homologous for each protein, (ii) a Multiple Sequence Alignment (MSA) was obtained with Muscle [41] and (iii) a position conservation score was calculated using internal programs counting the conservation of each amino acid for every sequence position. This conservation index allows identifying positions that may play an important role in function. A search for co-evolving residues was also performed using Freecontact [42] in Evfold mode [43] since it combines a fast implementation of two existing methods and yields amongst the best results for membrane proteins [43,44]. Default parameters were used for processing this analysis that may bring helpful information to guide further experiments when no experimental 3D structure is available [27].

### Family annotations

The domain decomposition was computed using InterProScan [45]. The domains are displayed as an interactive Scalable Vector Graphics with a description or boundaries when the user positions the cursor on a domain. The InterProScan output contains external hyperlinks for domain definitions, as well as dedicated ontologies predictions. The SVG was processed to trigger a query within the RESPIRE database when possible or to link to the upstream description otherwise.

### Structural information, annotation and model prediction

Structural annotations for each protein entry (**target**) were initially extracted from UNIPROT. Since PDB numbering can be different from the protein sequence in UNIPROT, each PDB is split by chains and renumbered to UNIPROT numbering using ProDy [46] to ensure an unambiguous mapping of UNIPROT features. When the 3D structure encompasses a large part of the target sequence, *i*.*e*. a large structural coverage (see below), the secondary structure assignment of the most representative structure is analysed with DSSP [47]. When the structural coverage is too low, the secondary structure prediction was done with PSIPRED [48].

In the absence of a 3D structure for the human form, the homology method was applied. It assumes that similar sequences share a similar 3D fold even when proteins share a very low sequence identity percentage. This property has led to identifying so-called protein superfamilies. Hence, we searched for homologous sequences having an atomic 3D structure available (called “**templates**” in the following) in Protein Data Bank (PDB, [49]) using Blast software or HHblits, a highly sensitive similarity search method based on hidden Markov models [50]. Default recommended parameters were used in both cases. Results of search were classified into four categories that guided the choice of appropriate tools to establish 3D models.

The categories detailed in **Table 2** are based on (i) the coverage on the target sequence (%cov), which is defined as the percentage of amino acids aligned between the template and the target sequences, (ii) the sequence identity (%id) between the target and the template sequence once aligned and (iii) the number of experimental structures needed to obtain a complete model.

**Table 2:** Category of 3D structural models and tools.

	Coverage (% cov)	Sequence identity (%id)	Number of templates	Tools	Secondary Structure
Experimental	> = 80%	~100%	1	Structure as is	DSSP [47]
Comparative modelling	60%< %id < 80%	25% < %id < 100%	≥ 1	Modeller/Medeller	PSIPRED [48]
Fold recognition	< 60%	% id < 25%	≥ 1	I-Tasser	PSIPRED
ab initio	0	0	0	Rosetta	PSIPRED

Thresholds were adapted to account for the differences in sequence characteristics between soluble and membrane proteins due to the different environment in which they are embedded. For comparative modelling [51], the thresholds were %id >35 and %cov >70 for soluble proteins, while lower thresholds are applied, %id>25 and %cov>60 for membrane proteins [52]. When the structural coverage was lower, a threading approach (alternatively called fold recognition) was considered to detect far putative homologous template. When no template could be used to predict the protein structure (no structural coverage), *ab initio* fragment-based assembly method was applied. For soluble proteins or single membrane-spanning proteins, MODELLER software [53,54] was used for the comparative modelling category, while MEDELLER was used for multiple membrane-spanning proteins [55]. For the threading category, I-TASSER [56] was used without distinction between soluble and membrane proteins. For *ab initio* category, Rosetta [57] and Rosetta membrane [58] were primarily used. For model production, each method was executed with default options. The best model produced was determined for every software by using its internal scoring function: objective function for MODELLER, cscore for I-TASSER, lowest energy for ROSETTA. Only the best model from each method according to their internal scoring function is displayed in RESPIRE. This model evaluation shall be enriched with independent scoring functions [59]. Each model can be downloaded for visualization in PyMol [60]. Depending on the protein modelling method, the prediction can take up to one week to be performed.

### Description, implementation and architecture of the RESPIRE database

The database is stored in MySQL version 5.5.32 and tables where created using the ORM implementation as available in the DJANGO framework. Data analysis, parsing and import were performed with *in-house* routines based on bioperl [61] or biopython [35]. The project development was managed using the SCM git and REDMINE. The web server is powered by DJANGO in WSGI mode under Apache 2.4 in Ubuntu 14.04 LTS, the responsive design is obtained using Bootstrap (http://getbootstrap.com/), jQuery (http://jquery.com/) and BioJS [62]. Interactive structure visualization is offered to the user using JSMol [63]. Interactive sequence-to-structure mapping is performed using JSAV [64].

The database is architected around 10 main tables filled in into two steps (*see above*). Data gathering is performed regularly from upstream sources according to their specific release schedule and updated monthly in RESPIRE (**Table 3**). In addition to the upstream databases stored locally, each protein contains on average 35MB of processed data and predictions, for a total of 80GB. Altogether the computing time dedicated to add structural information and predictions on the database represents multiple months on a dedicated cluster of 200+ cores.

**Table 3:** Schedules for data queries from reference databases, data processing and structural model prediction.

Source and URL	Information	Upstream schedule	RESPIRE query	Method	RESPIRE update
UNIPROT, https://www.uniprot.org/	Identifier, protein name, protein description, sequence, molecular weight, refseq and pdb identifiers	Monthly*	One week following uniprot update	Automatic with manual validation	monthly
PDB, https://www.wwpdb.org/	pdb files (cif, fasta and pdb format)	Daily	Synchronised with UNIPROT update	Automatic	Monthly **
OMIM, https://www.omim.org/	Description entries	Daily	Synchronised with UNIPROT update	Automatic	Monthly **
Biogrid, https://thebiogrid.org/	Interactions	Daily	Synchronised with UNIPROT update	Automatic	Monthly **
Gene Ontology, http://www.geneontology.org/	Annotations	Daily	Synchronised with UNIPROT update	Automatic	Monthly **
Interpro, https://www.ebi.ac.uk/interpro/	Domains	Every two month***	Semestrial	Manual	On-demand
ISBT, http://www.isbtweb.org/	Reference antigen definition	ISBT consortium	As required	Manual	
Models	N/A	N/A	Year	Automatic with manual validation	On-demand

* there is not update on uniprot entries in July.

** the update is processed after the initial UNIPROT update

*** Estimated from existing InterPro releases

## Results and discussion

The interest of our approach comes from the integration and automated mapping of protein data related to RBC. The user can find in a single database the protein expression level for a given cellular differentiation stage, proteins related to erythrocyte diseases and for each protein a structural status deduced from crystallography or NMR experiments. A strong effort was put to produce three-dimensional models using state-of-the-art methods, whose reliability depends on the structural content available.

### Protein content and selection

The red blood cell needs many differentiation steps to reach the mature, concave-like, nucleus void, cell also called normocyte or erythrocyte [1,2]. In blood, alongside erythrocytes, there are also pre-matured erythrocytes, the reticulocytes, both present as circulating cells. These two RBC are difficult to isolate one from the other [3]. Furthermore, even after careful examination, mass spectroscopy methods may over detect some proteins within the cells because trace of peptides can be present in reticulocytes without being present as a whole and functional protein [3]. One example is the presence of nuclear proteins for normocytes and reticulocytes where they should be no more detectable [3]. Due to these limitations, we chose to use the protein content reviews from the literature as the reference of existing knowledge and we will refine the protein content for every differentiation stage, as new data will permit.

### Database statistics

The database contains 2475 proteins for an average sequence length of 380 amino acids. On average, each protein has 4 sequence variants, and binds to more than 30 other proteins (not all incorporated in RESPIRE). Half of proteins have (partial) experimental structural data and more than a quarter of proteins have a model produced exclusively for RESPIRE. More than 1800 diseases are linked to RBC proteins from 3100 OMIM entries. Nearly 8500 gene ontology functions divided in three classes allow to regroup proteins (biological process, in a cellular component or possessing a particular molecular function). More than 380 proteins are annotated as integral or single-pass transmembrane proteins. These statistics are regularly updated and presented in detail on the “Statistics” tab in RESPIRE, with dedicated links to each category to restrict the protein search. According to the structural content available for each protein, 376 proteins were classified in the experimental category (no prediction is needed since there is sufficient structural data available), 595 proteins belong to the comparative modelling category, 496 proteins belong to the threading category and 306 do not possess any structural content and therefore need to be modelled using *ab initio* methods.

### Information available in RESPIRE for each protein

**Fig 1:**
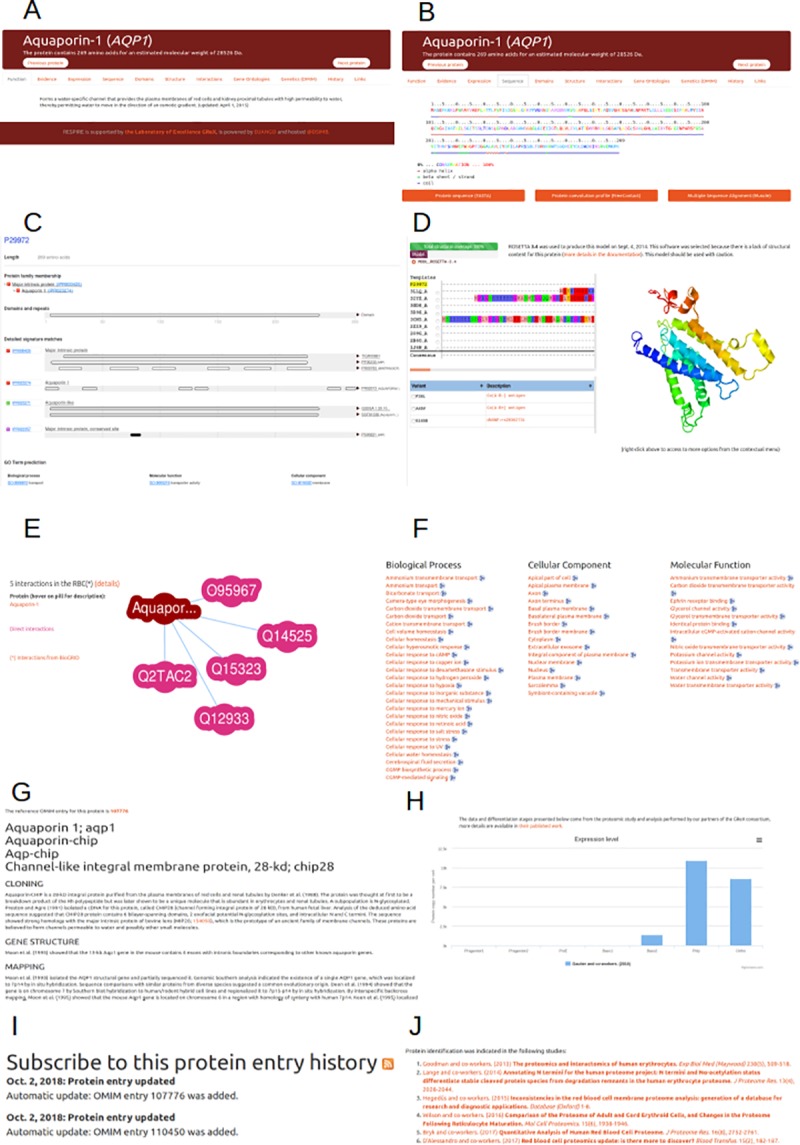
Description of the enriched protein entry available in the RESPIRE database. Aquaporin-1 protein (RESPIRE id: 641) serves as an example. (A) Protein name, amino acids length and molecular weight, as parsed from Uniprot. (B) Sequence details enriched with conserved position in the protein family, secondary structure representation in cartoon and amino acids co-evolution, color-coded from low conservation (blue) to high conservation (red) (see text for details). (C) Protein domains decomposition generated using InterProScan. (D) Protein structure interactive visualisation allowing to map the position of variants and mutants on the displayed molecule. (E) Binding partners mapping, with links to the corresponding entry in the database or to UNIPROT when required. (F) Gene Ontology annotations with links to all proteins related to this term in the database, and links to the Gene Ontology web site when the user wants to complete the definition. (G) Additional clinical and genetic information concerning the protein, as gathered with permissions from the OMIM database. (H) Profile of the protein profile expression during hematopoiesis. (I) History of the protein entry, with a complete trace of updates. (J) Indication of the protein detection in scientific literature.

For a given protein entry, the first tab displays the protein function as annotated in UNIPROT and when this information was updated in UNIPROT (**Fig 1A**).

The sequence frame contains the protein sequence enriched with a color-coded conservation index (from blue, low conservation, to red, high conservation), and the DSSP secondary structure [47] assignment or PSIPRED prediction [48]. This conservation index was computed after the multiple alignments of related sequences found by BLAST. As classical sequence alignments can fail in determining the underneath importance of specific amino acids for the protein structure or function, especially if they are mutated in tandem, a co-evolution study was performed. Due to their file size, the complete sequence processing (Blast search, Multiple Alignment) cannot be visualized in RESPIRE, so download buttons are available for the sequence (UNIPROT), the co-evolution profile and the multiple sequence alignment using muscle (**Fig 1B**). A membrane annotation is indicated if this protein is either referenced (i) as a Single-Pass or Multiple-Pass protein in UniProt, (ii) as a plasma membrane protein in Gene Ontology, or (iii) if the TOPCONS prediction has detected at least one transmembrane segment.

In the domains tab, the protein decomposition into subdomains performed using InterProScan is displayed. Subdomains are assembled into coloured sections with hyperlinks to their corresponding families in InterPro [45] and hovering on each coloured bar indicates the domain limits (**Fig 1C**).

**Fig 2:**
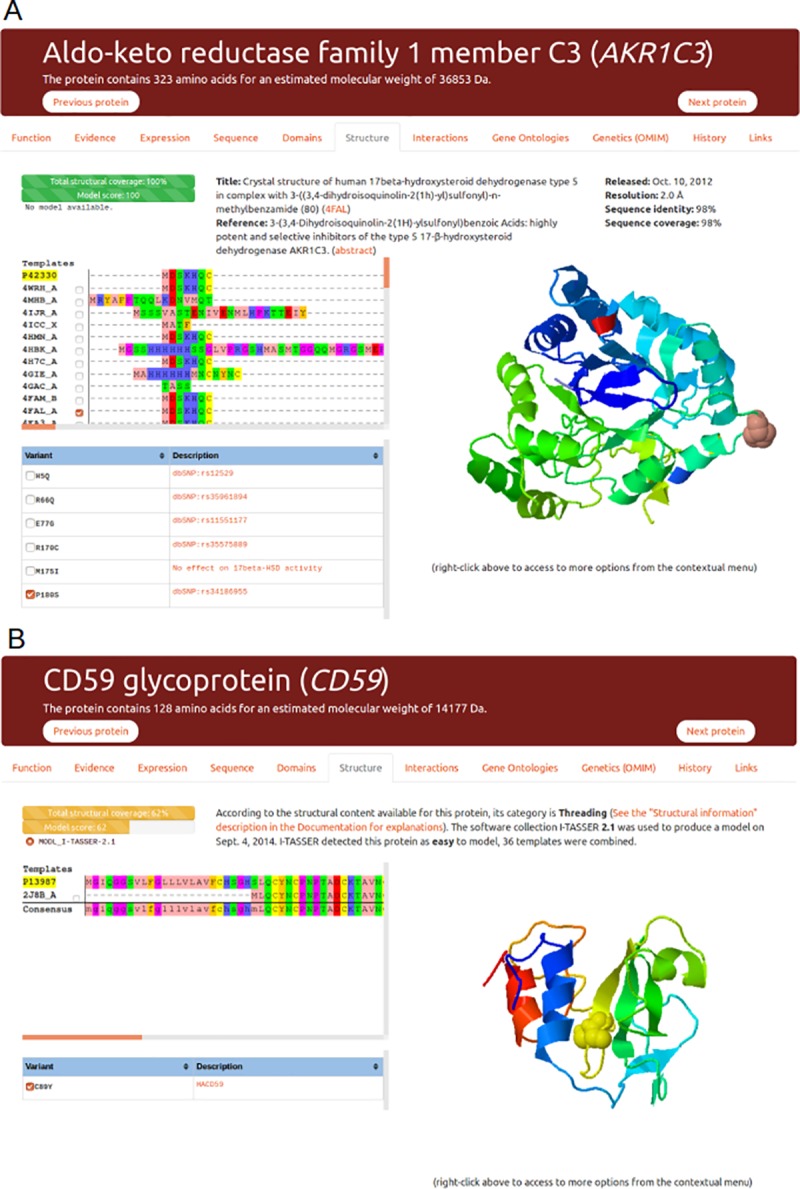
Details of protein structural content. Each experimental structure or produced model is displayed at the top with a score to rank the more complete structures: first for experimental structures, and then the best model according to the modelling method internal score. Hovering on each entry name shows in a condensed view the experimental information or the model origin. By clicking on any entry presented, the corresponding PDB file is uploaded to the JSmol viewer for interactive manipulations. The more complete JSmol menu can be opened with a right-click on the JSmol canvas. A list of known variants is shown below the templates window, with a brief description and a link to the upstream description. By selecting a variation, the user will highlight on the structure the position of the amino acid mutation. (**A**) Detail of the structural interactive view for Aldo-keto reductase family 1 member C3. The mutation of a proline for a serine at position 180 is represented in brown sphere representation. (**B**) Detail of the CD59 glycoprotein entry. For this protein, there is no sufficient structural coverage available (indicated in orange, 62% only can be determined using structural experimental data), so a model was produced using I-TASSER [56,69,70]. This resulting model is considered an average model with a TM-Score of 0.62.

When either a protein structure or a model is available, the structure frame presents the three-dimensional coordinates in an interactive window (**Figs 1D and 2**).

It is important to visualize the location of known natural variations or mutations to assess qualitatively their impact on the protein structure. These interactions can be directly mapped on the structure by clicking on the dedicated checkbox. If no positions are described in the UNIPROT upstream entry, no list is provided to the user.

RBC proteins may interact with many proteins to fulfil their function; these interactions are assembled from various tissues in BioGrid [39]. To integrate these interactions into a functional network, the Interactions tab shows a dynamic responsive graph containing the direct binding partners available only in the RBC, limited to the first 50 members of the network for performance issues (**Fig 1E**).

Many inferences between proteins are derived automatically from data-driven knowledge associations as performed by the Gene Ontology Consortium [65,66]. This information is regrouped under the Gene Ontology (GO) frame, which allows browsing the database content alternatively by clicking on a GO category. From one protein card, it is possible to retrieve all proteins similarly involved in a specific biological process, in a cellular component or possessing a particular molecular function (**Fig 1F**).

According to their prevalence in populations, the knowledge concerning some RBC diseases such as sickle-cell disease is widespread in the community [67]. For more specific disease such as malaria [17,68] or for the determination of new blood group antigens [15], this knowledge is harder to acquire. The knowledge presented in the Genetics tab embeds an extract of the OMIM entry for a protein, with a link to the complete entry in the OMIM web site. This OMIM entry is also processed to offer links to proteins in RESPIRE for RBC diseases or to the upstream OMIM entry when these diseases are found in other tissues (**Fig 1G**).

The data presented in our database mainly present the content of the latest stages of the RBC differentiation. In order to get a broader overview of the protein expression levels during the RBC maturation process, an interactive diagram is available under the Expression tab (**Fig 1H**). These expression data were carefully determined by our partners of the GR-Ex consortium [12].

Many entries in the database come from external databases, it is therefore important to track the changes appearing in the protein card. The History tab references all the modification of the protein entry in the database. It is also possible to register to the protein feed using tools such as Feedly (https://feedly.com) or Reeder (https://reederapp.com/) to be informed rapidly when a modification happened (**Fig 1H**).

### Structural annotation and model prediction

In comparison with other databases dedicated to RBC proteins, an important interest of our database relies on the important information brought by the 3D structure and the possibility of visualizing experimental structures or models produced specifically for RESPIRE. When available, experimental PDB structures [43] are presented, but most of the time, these structures are missing for the human species. We therefore computed the existing experimental structural coverage to qualify the category for modelling protein structures (see methods). The proteins belonging (i) to the comparative modelling category were modelled using Modeller for soluble proteins [53,54] or Medeller for membrane proteins [55]; (ii) to the threading category were modelled using I-TASSER [56,69]; (iii) to the ab initio category were modelled with ROSETTA suite [57]. The membrane content could be considered within ROSETTA using a dedicated protocol [58]. This focus on membrane proteins is particularly important since some of them have a high therapeutic interest but they are much less characterized experimentally than globular proteins. To ease a better comprehension of these categories and to provide an estimation of the model quality, two progress bars are displayed in the Structure tab.

This condensed view allows also interactively to display and retrieve a PDB file or the model produced for RESPIRE. When the user clicks on a pdb entry, its title and reference are indicated, when a model is selected, the details of its prediction are provided (**Fig 2**).

### Querying the database

There are many ways to query features in the database. The general principle is to provide many hyperlinks redirecting within RESPIRE when possible or to the upstream annotation otherwise. For some annotations, an additional link to an icon directly allows the opening of the upstream reference in a new window.

**Fig 3:**
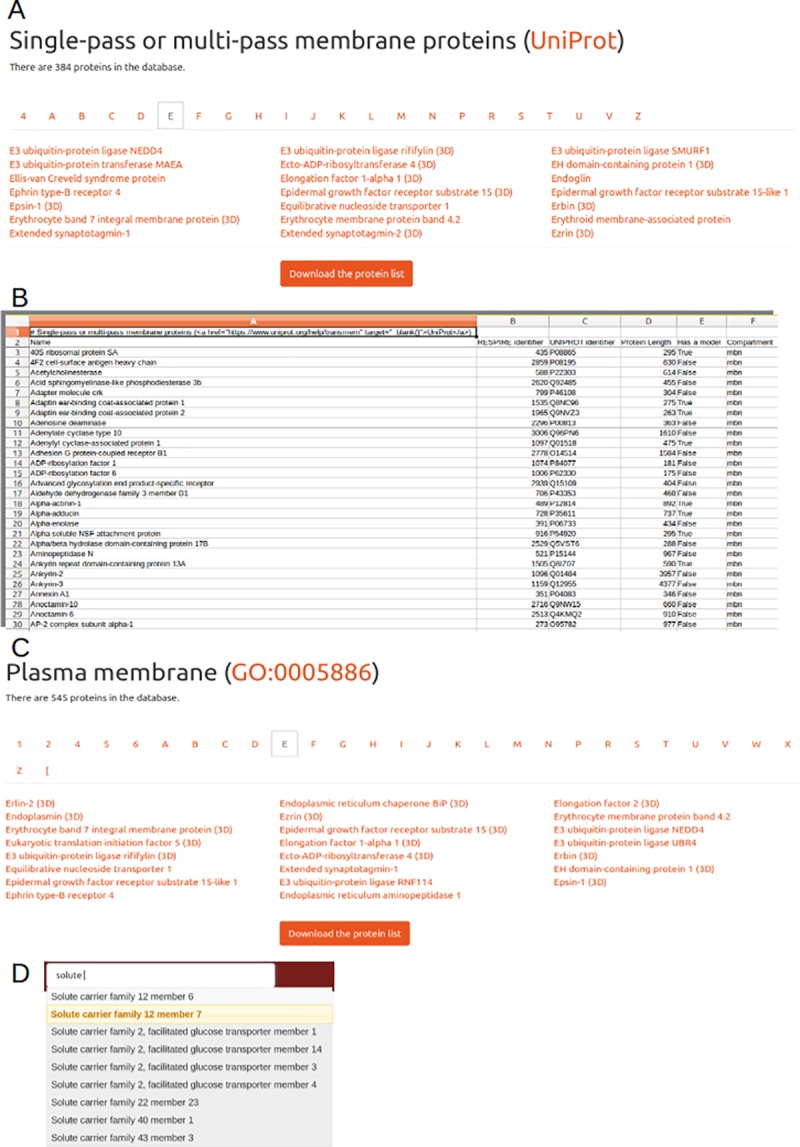
RESPIRE can be accessed through various queries. **Example of precomputed requests**. Clicking on the links will retrieve all proteins pertaining to a given category. (**A**) Query results concerning the “membrane” annotation in UNIPROT. 384 proteins are annotated with this membranous localisation. By clicking on a number or letter, the user can see all the protein names starting with this item, with an indication of an existing structural when the (3D) keyword is appended to the name, proteins starting with letter E are displayed. (**B**) By clicking on the button “Download the protein list”, the user will be provided a Comma Separated Value file containing the protein name, its identifier in RESPIRE and UNIPROT, the protein length, its structural status and its cellular localisation. (**C**) A close “plasma membrane” annotation can be accessed by clicking on the GO entry GO:0005886 in the statistics tab or in any entry possessing this annotation. This time 545 entries will be displayed and can also be retrieved. (**D**) To access rapidly to a protein using its name or description, it is possible to use the “search-as-you-type” facility provided at the top right in the menu, for instance to retrieve a member of the Solute Carrier Family.

Complex queries are also available in a dedicated menu for specific categories, namely proteins with a model (RESPIRE), proteins at the cell membrane (as defined in UNIPROT), protein defining blood group antigens (ISBT) or proteins associated to a disease (OMIM) (**Fig 3A**). The results of these queries can be retrieved in Comma Separated Value (CSV) format for further processing off-line in any spreadsheet editor (**Fig 3B**). It is also possible to browse the database by clicking on hypertext links provided at various places, like for instance clicking on a given gene ontology category under the Genetics tab to retrieve all proteins involved in a specific process (**Fig 3C**). It is also possible to use the “search as you type” box to search by UNIPROT accession id or protein name (**Fig 3D**).

For more complex queries, a more advanced form allows the combination of criteria. In this form, selecting (a) **Cell Localization** “Single-pass or Multiple pass membrane proteins (Uniprot)”, (b) **Protein name** “transport” and (c) **Protein Size (AA)** “200–800”, there will be 14 answers in RESPIRE related to a protein containing the “transport” keyword in its name without models in the RBC membrane. Again, this query result can be downloaded as a CSV file.

These multiple search strategies are important to retrieve a protein list concerning close but not overlapping queries. As an example, the membranous status of a protein is complex: (i) many proteins can have a transient membranous contact for maturation or trafficking within the cell, (ii) even by limiting only the definition of membranous proteins to the cell membrane localization, it is still possible to classify a protein as an integral or as a single-pass membrane protein. This complexity of membrane definition including localizations and/or organisations is assembled differently in different databases, giving different results for close queries. For instance, for membrane proteins, 384 proteins are membranous in RESPIRE if the “single-pass or multiple-pass membrane” annotation of UNIPROT is considered while in Gene Ontology, the “plasma membrane” annotation (GO:0005886) leads to 545 entries. The possibility to address both queries in RESPIRE is the best way for tackling these difficulties in a flexible manner.

### Contact and expert annotation

RESPIRE is a combination of upstream data references and of specifically produced protein models. Our main objective is to propose a high quality of service with regularly updated information. Importantly, any expert in RBC is welcomed to contribute in RESPIRE evolution and update. In this perspective, a contact form with pre-defined subjects is available if a user is willing to further enrich a given protein entry or report problems. The “Collaboration” subject is dedicated to more complex demands such as the incorporation of new data in the database, the additions of links to or the processing of other reference databases. The “Feature Request” is mostly for database updates on specific subjects like the addition of pre-defined queries, demands for updating a given protein model. The “Bug report” subject is mostly to pinpoint specific problems for a given entry. If no demand can be classified with previous subjects, the “General” subject is open to any remark or contribution. Each request will be processed regularly, and responses to demands should be answered within a week. Depending on the amount of work required, these demands will be answered rapidly or shall be incorporated in the next RESPIRE release.

## Conclusion and future directions

The importance of RBCs in vital processes has driven the extensive characterization of protein abundance and expression level using large-scale studies. Up to now, it is difficult to assemble protein information linking these experiments and reference databases. To address these needs, we have set up a new database called RESPIRE devoted to red blood cell proteins, starting from a list of proteins available from the literature. The RESPIRE database combines sequence, structure and functional annotations altogether with original data obtained using up-to-date bioinformatics methods [17,56–60,69,71–72] in particular 3D models supported or not by experimental data [73]. These predicted models should be considered carefully, but can serve as tools to design further experimental validations. As new structural information is available regularly, low-quality models will be regularly improved.

In the future, we shall continue to enrich this database with experimental results as they become available in the literature but also with manual curation involving biologist members of the research consortium GR-Ex. This curation effort is expected to focus on inherited disease such as sickle-cell disease or Diamond-Blackfan anemia, or to focus on infectious diseases such as malaria. We will expose convenient access to these data to ease cross-referencing in more general-purpose databases.

The database is freely accessible, with an unrestricted access to data for download and external analysis. The evolution of the database is detailed per protein in a dedicated tab, and globally in a dedicated page.

## Abbreviations

RBC: Red Blood Cells
RMSD: Root Mean Square Deviation
PDB: Protein Data Bank
